# Mesoporous Silica Platforms with Potential Applications in Release and Adsorption of Active Agents

**DOI:** 10.3390/molecules25173814

**Published:** 2020-08-21

**Authors:** Cristina Chircov, Angela Spoială, Cătălin Păun, Luminița Crăciun, Denisa Ficai, Anton Ficai, Ecaterina Andronescu, Ștefan Claudiu Turculeƫ

**Affiliations:** 1 Department of Science and Engineering of Oxide Materials and Nanomaterials, Faculty of Applied Chemistry and Materials Science, University Politehnica of Bucharest, Spl. Independentei 313, 060042 Bucharest, Romania; cristina.chircov@yahoo.com (C.C.); angela.8317@gmail.com (A.S.); catalinpaun13@gmail.com (C.P.); anton.ficai@upb.ro (A.F.); ecaterina.andronescu@upb.ro (E.A.); 2 Department of Inorganic Chemistry, Physical Chemistry and Electrochemistry, Faculty of Applied Chemistry and Materials Science, University Politehnica of Bucharest, 1-7 Gh Polizu Street, 050054 Bucharest, Romania; luminita.craciun@upb.ro; 3 Academy of Romanian Scientists, Ilfov st. 3, 050054 Bucharest, Romania; 4 Department 10. General Surgery, Faculty of Medicine, Carol Davila University of Medicine and Pharmacy, 8 Eroii Sanitari, Sector 5, 050474 Bucharest, Romania; claudiu.turculet@umfcd.ro; 5 Surgery Clinique, Emergency Hospital Floreasca Bucharest, 8 Calea Floresca, 014461 Bucharest, Romania

**Keywords:** mesoporous silica nanoparticles, synthesis, functionalization, biomedical applications

## Abstract

In recent years, researchers focused their attention on mesoporous silica nanoparticles (MSNs) owing to the considerable advancements of the characterization methods, especially electron microscopy methods, which allowed for a clear visualization of the pore structure and the materials encapsulated within the pores, along with the X-ray diffraction (small angles) methods and specific surface area determination by Brunauer–Emmett–Teller (BET) technique. Mesoporous silica gained important consideration in biomedical applications thanks to its tunable pore size, high surface area, surface functionalization possibility, chemical stability, and pore nature. Specifically, the nature of the pores allows for the encapsulation and release of anti-cancer drugs into tumor tissues, which makes MSN ideal candidates as drug delivery carriers in cancer treatment. Moreover, the inner and outer surfaces of the MSN provide a platform for further functionalization approaches that could enhance the adsorption of the drug within the silica network and the selective targeting and controlled release to the desired site. Additionally, stimuli-responsive mesoporous silica systems are being used as mediators in cancer therapy, and through the release of the therapeutic agents hosted inside the pores under the action of specific triggering factors, it can selectively deliver them into tumor tissues. Another important application of the mesoporous silica nanomaterials is related to its ability to extract different hazardous species from aqueous media, some of these agents being antibiotics, pesticides, or anti-tumor agents. The purpose of this paper is to analyze the methods of MSN synthesis and related characteristics, the available surface functionalization strategies, and the most important applications of MSN in adsorption as well as release studies. Owing to the increasing antibiotic resistance, the need for developing materials for antibiotic removal from wastewaters is important and mesoporous materials already proved remarkable performances in environmental applications, including removal or even degradation of hazardous agents such as antibiotics and pesticides.

## 1. Introduction

With 37 wt.%, silica is the most ubiquitously found oxide on earth and it represents the general name for silicon dioxide-consisting inorganic ceramic materials. The tetrahedral structure is formed by the strong covalent bonding between the silicon atoms and the surrounding four atoms of oxygen, which leads to the formation of hard materials. However, the silica atoms can assemble into a great variety of arrangements owing to the flexibility of bridging between the constituent atoms. Silica can occur naturally, or it can be synthetically produced within a wide range of structures, from entirely amorphous, which has the property of retaining the memory of its path owing to its high viscosity and low diffusivity, to highly crystalline, such as quartz. Moreover, silica materials can be porous, non-porous, anhydrous, or hydroxylated, with the degree of hydration being proportional to the specific surface area, with the larger surface area meaning higher defect areas, which can be easily turned into silanol groups [1,2].

According to IUPAC, porous materials can be described based on the availability of the pores to an external fluid, namely, closed, open, blind, or through pores, as well as their shape, namely, cylindrical, ink-bottle, funnel-shaped, or slit-shaped pores [3,4]. The silica-based materials considered in this review are non-porous silica nanoparticles, mesoporous silica nanoparticles (MSNs), mesoporous silica-based materials, and biosilica. They can be obtained at low temperatures, which makes them suitable for biomedical applications [5]. The pore size leads to the classification of silica materials into microporous, with a pore diameter smaller than 2 nm; mesoporous, with diameters in the range of 2 and 50 nm; and macroporous, with pores larger than 50 nm [6,7]. The focus of this review is mesoporous silica, which will be thoroughly described further.

It is safe to assume that the field of mesoporous materials has been established owing to the considerable advancements of the characterization methods, namely the electron microscopy methods. They have allowed for the visualization of the pore structure and the materials encapsulated within the pores, enabling the understanding of these materials along with the X-ray diffraction methods and specific surface area determination by the Brunauer–Emmett–Teller (BET) technique [8].

Silica-based mesoporous nanomaterials are the most widely exploited in their field [9]. The synthesis of spherical mesoporous silica particles through the Stöber method was first reported in 1997 by Unger et al. [10]. Further research has led to the downsizing of the mesoporous silica to the nanoscale, allowing for the development of novel applications [11]. MSN are solid materials with purely inorganic siloxane frameworks and an ordered, porous structure consisting of a multitude of empty channels that can absorb or encapsulate large amounts of bioactive compounds [12,13].

To talk about mesoporous silica’s history, we must look back in the mid 1990s, where Mobil Oil Corporation from the United States and Kuroda from Japan uncovered materials with larger pores than zeolites. It is worth mentioning that the first article regarding mesoporous silica applications in drug delivery using MCM-41 (Mobil Composition of Matter No. 41) was presented in 2001 and had considerable importance within the biomedical field. Investigating MSN deeply, the findings concluded that functionalizing them with different materials could allow for the administration into the blood stream for targeting specific sites of the tumor tissues. To improve MSN functionalization, core-shell structures were developed with perspectives in medical imaging; for instance, magnetic nanoparticles embedded into silica shells have been successfully effective in magnetic resonance imaging (MRI) and magnetic drug targeting [14].

MSNs have been studied for a variety of applications, from nanoreactors for industrial catalytic reactions [13], to adsorbents, biosensors, drug delivery systems, gene carriers, phototherapy, and tissue engineering [13,15,16]. However, most studies focus on their use as drug nanocarriers, as they meet all the requirements, namely, biocompatibility, high loading capacity, no premature release, specific targeted delivery, and controlled release for an effective local drug concentration [17,18]. In the biomedical field, the hosting and delivery ability is especially exploited, while in the environmental field, the adsorption ability is especially exploited for the removal of hazardous agents from nature (including drugs and their metabolites such as antibiotics). Furthermore, there are physical and chemical strategies for MSN surface modification, which might enhance the specificity of delivery and the rate of adsorption and release [19]. Surface modification is especially important because it can be exploited to modulate adsorption and release characteristics by changing the strength of the interaction between support and active agents.

As MSN can damage the integrity of biological membranes through the prolonged circulation time, biodistribution, and interactions with the surrounding cells, the in vivo toxicity and behavior must be accurately determined and adjusted to meet the requirements for biomedical applications [20].

The purpose of this review is to review the methods of MSN synthesis and derived characteristics, the available surface-functionalization strategies, and the most important applications of MSN in biomedical and environmental applications.

## 2. Characteristics of Mesoporous Silica Materials

Mesoporous silica materials have attracted a great scientific interest in various fields owing to their unique characteristics. Structural properties include uniform and tunable pore size (typically within the range of 2–6 nm), uniform and tunable nanoparticle size (50–200 nm) and shape, possibility of surface functionalization, high surface area (700–1000 m^2^/g), large pore volume (>0.9 cm^3^/g), and control over the structure of the pore networks and the gating mechanism of pore openings [9,15,16]. Moreover, these nanocarriers are resistant to pH, heat, mechanical stress, and hydrolysis-induced degradations; have bifunctional surfaces, as they possess an internal surface and an external surface; and their fabrication is simple and cost-efficient [9,12].

According to the method of synthesis and its characteristics, MSN can be synthesized in a variety of forms (Table 1). MCM-41, developed by Mobil Crystalline Materials or Mobil Composition of Matter, is one of the most widely designed MSNs, with a hexagonal layout and a pore width between 2.5 and 6 nm. Other MSNs from the MCM category are MCM-48 and MCM-50, with cubic and lamellar arrangements, respectively. Subsequently, the Santa Barbara amorphous type materials, including SBA-11 (cubic), SBA-12 (3D hexagonal), SBA-15 (hexagonal), and SBA-16 (cubic-cage structures), which have larger pores and thicker silica walls, have been synthesized by the University of California, Santa Barbara. Furthermore, other synthesized MSN are FSM-16 (folded sheets of mesoporous materials); TUD-1 (Technical Delft University); HMM-33 (Hiroshima Mesoporous Material); COK-12 (Centrum voor Oppervlaktechemie en Katalyse/Centre for Research Chemistry and Catalysis); and FDU-2, -11, -12, -13 (Fudan University) [15,21,22]. Additionally, MSN can also be synthesized as hollow and rattle-type nanoparticles, which are interstitial hollow-spaced, mesoporous shells with low density and high surface area [21].

**Figure 1:**
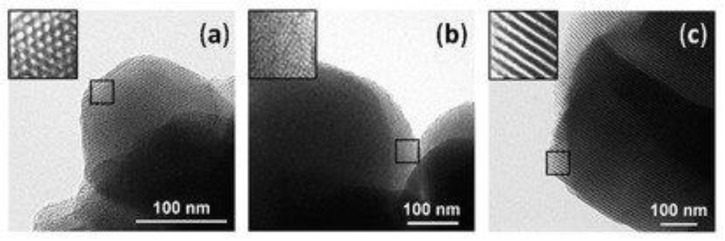
Transmission electron microscopy (TEM) images for calcined Mobil Crystalline Materials or Mobil Composition of Matter (MCM)-41 (**a**), MCM-48 (**b**), and Santa Barbara amorphous (SBA)-15 (**c**). Reprinted from an open access source [40].

Among the previously mentioned mesoporous silica materials, MCM-41, MCM-48, and SBA-15 are the most commonly used for adsorption and release purposes in drug delivery and environmental applications. While at the atomic level, their structure is amorphous, they exhibit a highly ordered mesostructure at the molecular level. Their pores are in the form of channels, either longitudinal with a hexagonal cross-section for MCM-41 and SBA-15, or tridimensional, creating cross-sections in all three directions of the structure (Figure 1) [22].

## 3. Mesoporous Silica Synthesis Methods

Generally, mesoporous silica materials synthesis requires the addition of surfactants as structure directing/template agents. The surfactants will undergo supramolecular assembly by self-aggregating into cylindrical micelles at concentrations higher than the critical micelle concentration, which will lead to the condensation of the silica precursors at the surface. Subsequently, the surfactant template will be removed through calcination or solvent extraction in order to generate the pore network [41,42]. Mesoporous silica materials can be synthesized through various routes, including sol–gel and aerogel methods, precipitation, hydrothermal method, layer-by-layer self-assembly, chemical etching technique, microwave-assisted technique, spray drying, and template-directed method [41,43,44].

Stöber, Fink, and Bohn first reported the synthesis of colloidal spherical silica particles in their pivotal study in 1968 [15,45]. Nowadays, the use of ammonia-catalyzed hydrolysis of silicon alkoxide and the consequent condensation of the silanol monomers obtained is generally known as the Stöber method [45,46]. The Stöber method is a sol–gel process, which is a wet chemistry technique to produce solid materials starting from small molecules (surfactants and silica precursors) by a bottom-up approach.

The formation of silica particles through sol–gel chemistry involves two main mechanisms of reaction. Firstly, a silicon alkoxide, which serves as the precursor for silica particles, is introduced in a water/alcohol solution. The reaction between the silicon alkoxide Si (OR)_4_ and the water molecules will generate Si-OH groups through a hydrolytic process. Subsequently, as the hydroxyl groups act as reactive sites, they will further react with other (partially hydrolyzed) precursor molecules through the condensation mechanism. Therefore, oxo bridges (–O–) between silicon atoms will form and will generate the growth of the silica particles [45,47,48]. The most widely used silicon alkoxide as a precursor for silica, also described in the Stöber method, is tetraethoxysilane or tetraethyl orthosilicate (TEOS) and water and/or ethanol are typically used as solvents [41,49]. Additionally, tetramethoxysilane or tetramethyl orthosilicate (TMOS), tetrapropoxysilane or tetrapropyl orthosilicate (TnPOS), and tetraisopropoxysilane or tetraisopropyl orthosilicate (TiPOS) can be used as silica sources [48]. Moreover, the sol–gel process can occur under acidic or basic conditions, which will further influence the properties of the so-obtained materials [47,49]. The mechanism for silica formation in the acidic environment involves the protonation of the alkoxy groups O–R and the hydroxyl groups O–H by the acid catalyst and the subsequent deprotonation, where the leaving group is the alcohol R–OH. In the following stage, the –OH group of the partially hydrolyzed precursor is protonated, and further condenses with the –OH group of another partially hydrolyzed precursor molecule to form oligomers. By contrast, in a basic environment, the –OH group will directly attack the silicon atom [48]. The most common mechanism of obtaining mesoporous silica follows the above presented steps and protocol, but the addition of the silica precursor occurs after the cylindrical micelles formation. In this manner, the siloxane structure is generated onto the surface of the ordered rod-like micelles (Liquid Crystal Templating Mechanism). Mesoporous silica can also be obtained by three additional mechanisms, namely, silicate rod assemble model, charge density matching mechanism, or cooperative organization mechanism [50,51,52].

The characteristics of the particles can be influenced by the nature and concentration of the alkoxide precursor, which affects the rate of the hydrolysis and condensation reactions; the nature and the content of the solvent, as alkoxy group–water ratios; the pH; the temperature; and the presence of templates [47,48]. As the synthesis of MSN commonly involves the use of surfactants, numerous studies have been performed in regard to their influence on the shape and porosity of the particles. Therefore, cationic, anionic, and nonionic surfactants have been widely applied, and the molar ratio of the reaction mixture has been seen to greatly impact the particle parameters. Common surfactants used for MSN synthesis are the cationic cetyltrimethylammonium bromide (CTAB) and the triblock copolymers Pluronic P-123 and Pluronic F-127 [44]. For instance, in the case of MCM class, the most important factors affecting the formation of the distribution of the ordered pores are related to the ratio between the silica precursor, sodium hydroxide, and CTAB. According to this ratio, the formation of MCM-41, 48, or 50 is favored, while many other factors are additionally tuning the characteristics of these mesoporous materials.

### 3.1. MCM-41 Synthesis

**Figure 2:**
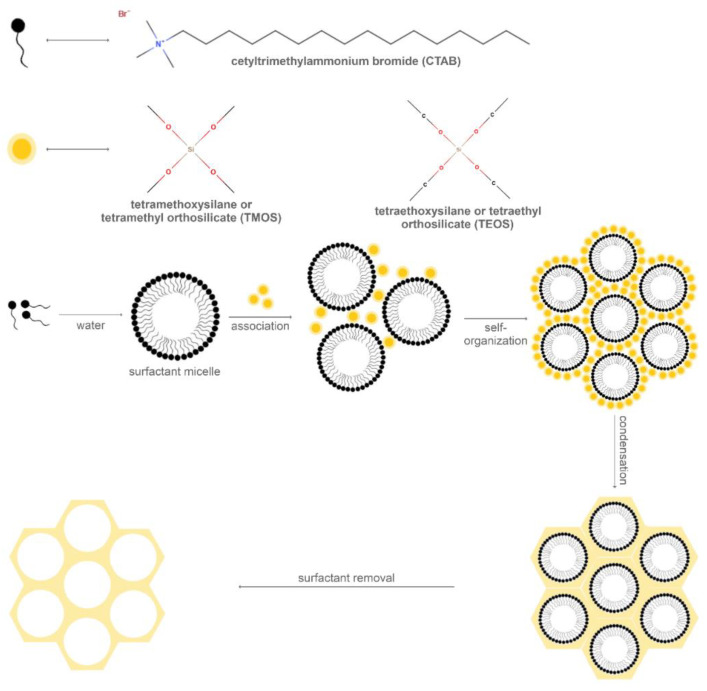
A schematic diagram of the processes involved in the synthesis of MCM-41.

The first report on MCM-41 synthesis involved the hydrothermal transformation of basic silicates and aluminosilicates in the presence of quaternary ammonium surfactants with various alkyl chain lengths. Specifically, the mesoporous silica material synthesis is based on the formation of surfactant cylinders and the subsequent aggregation of silica species to form a tubular structure through the polycondensation process (Figure 2). The conditions for silica precursor and surfactant mixing require a temperature in the range of 30–60 °C and an alkaline pH of 11. The mechanism is driven by the attraction between the positive charges of the cationic surfactant and the negative charges of the silica species. The silica precursor used for the original MCM-41 synthesis was TMOS, while the surfactant was CTAB. Additionally, TEOS or other sodium-silicates as silica sources; as well as ammonium salts with other counter ions, such as cetyltrimethylammonium tosylate and cetyltrimethylammonium chloride; or mixtures, such as CTAB and the amphiphilic Pluronic F68, could be used [53,54,55]. The properties of the nanoparticles, such as the homogeneity and the hexagonal shape, are considerably dependent on the temperature, the ratio between silica precursors and surfactants, and the rate of addition and agitation. The mesopores are generated by the removal of the surfactant, by calcination, treatment with ammonium nitrate, or surfactant extraction under reflux in acidified alcohol with hydrochloric acid, which will eliminate the electrostatic interactions between the surfactant and the silica [54].

However, as the conventional hydrothermal method requires at least 24 h, novel strategies based on the microwave-assisted hydrothermal method have been employed. In this manner, MCM-41 could be synthesized at 100 °C in 5 min. The optimal time, however, is 90 min, with properties of MCM-41 similar to the conventional method [55]. By contrast, compared with the conventional method, the use of ultrasound irradiation for mixture stirring led to the formation of MCM-41 nanoparticles with lower surface area and pore volume [56].

Furthermore, MCM-41 can be synthesized in the form of ropes, through the electrospinning technique. Recent works reported the formation of MCM-41 ropes in a basic environment using white wheat stem ash as a silica source, CTAB as the surfactant, and polyvinyl alcohol as the polymeric gelation agent. However, depending on the spinning voltages and distances, the mesoporous silica material can be obtained in the form of nanoparticles by electrospraying/electrospinning at 15–25 kV and 5–12 cm, and ropes, at 25–30 kV and 12–16 cm [57].

Novel strategies involve the synthesis of MCM-41 through green approaches in order to reduce the utilization or generation of hazardous materials. Therefore, silica materials from rice husk [58] or bagasse fly ash and sugar industry siliceous detritus were successfully used for the synthesis of MCM-41 via the sol–gel method [59].

### 3.2. MCM-48 Synthesis

**Figure 3:**
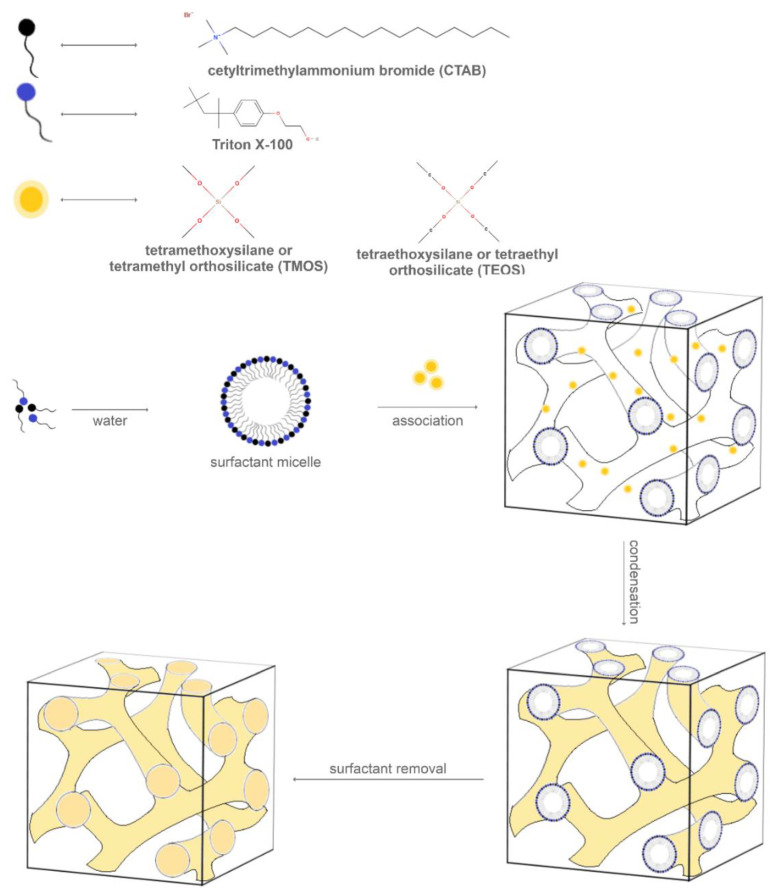
A schematic diagram of the processes involved in the synthesis of MCM-48.

The production of MCM-48 (Figure 3) requires more strict synthesis conditions, including high-purity materials, various surfactants, and highly basic environments for crystallization. Moreover, minor variations regarding the synthesis factors, such as silica source, temperature, alkalinity, and reactant ratio, might lead to the formation of MCM-41. Therefore, several studies have been performed for the establishment of specific conditions for obtaining MCM-48 [60].

One study reported the synthesis of MCM-48 using CTAB as the surfactant, TEOS as the silica source, ammonia or sodium hydroxide for adjusting the alkaline medium, and ethanol as solvent [61,62,63]. Furthermore, the influence of several parameters on the formation of MCM-48 structures was investigated. Specifically, the results showed that the washing step led to the formation of poorly ordered structures through a room temperature synthesis. Moreover, the drying conditions could also influence the structure of the mesoporous silica, as drying in an oven for 3 days at room temperature resulted in a highly ordered MCM-48 structure, whereas higher temperatures induced the partial formation of the hexagonal MCM-41 structure. Additionally, increasing the amount of water in the precursor solution led to the formation of MCM-41 structures by decreasing the surfactant micelle concentration. Similarly, an increased amount of ethanol in the reactant mixture induced the transformation from MCM-48 to MCM-41, as ethanol acted as a co-surfactant and not as a solvent [62].

Moreover, as MCM-48 forms through the association of silica and template micelle assemblies, its synthesis is highly dependent on the surfactant type. Hence, various surfactant types, including cationic surfactants, anionic surfactants, nonionic surfactants, neutral amine surfactants, block copolymer surfactants, and mixed surfactant systems, such as co-solute–surfactant, cationic–anionic surfactants, cationic–nonionic surfactants, and anionic–nonionic surfactants, have been studied for MCM-48 synthesis [64]. Therefore, studies reported the use of a combination of CTAB, a cationic surfactant, and Triton X-100, a nonionic surfactant, as a template to hydrothermally synthesize high-quality MCM-48 using fumed silica or rice husk ash as the precursor. The quality of the mesoporous silica material is considerably influenced by the temperature of the solution to which the fumed silica is added, the ratio between the two surfactants, and the crystallization time and temperature [60,65].

However, as the hydrothermal method is challenged by the long time required for the preparation of MCM-48 and the poor stability of the so-obtained structures [61,66], the incorporation of heteroatoms and the addition of crystal seed precursors, inorganic salts, or polyvinyl alcohol powder have been reported. Transition metals, including titanium, vanadium, iron, copper, and cerium, have been incorporated through various methods, such as the one-step hydrothermal method, template ion-exchange method, grafting method, and wet impregnation method, in order to construct catalytic active sites [66].

Additionally, the synthesis of MCM-48 through the pseudomorphic transformation of porous glasses has also been investigated. Porous glass granules and cetyltrimethylammonium hydroxide as the surfactant were used for the transformation into MCM-48 materials in Teflon autoclaves. However, this method might lead to the formation of bigger particles compared with the fine powders obtained through conventional methods [67].

### 3.3. MCM-50 Synthesis

**Figure 4:**
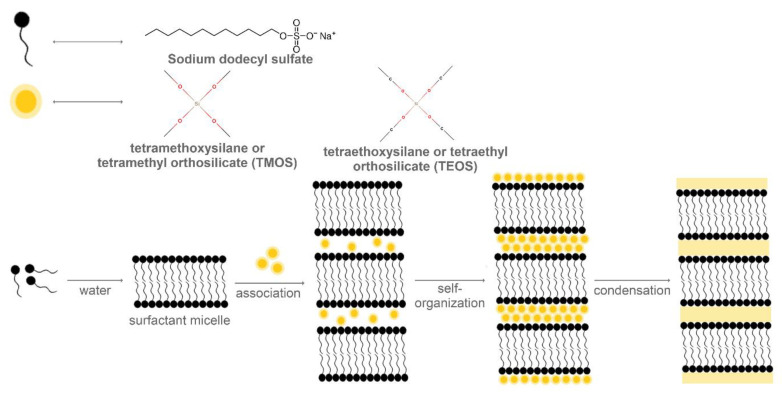
A schematic diagram of the processes involved in the synthesis of MCM-50.

As the thermal treatment of MCM-50 results in the collapse of the lamellar structure owing to the removal of the template (Figure 4), leading to a poor structural order and organization, its production and application is considerably limited [68]. One strategy to obtain the MCM-50 structure involves the use of Gemini surfactants, which have a binary structure with cationic and anionic parts. Thus, through the coupling of the counter ions exchange property, the final mesoporous structure can be modulated. The study reported the use of imidazolium-based Gemini surfactant, with an imidazolium head group and an ethoxyethylene spacer. In this manner, by coupling a long-chain dodecyl sulphate to the imidazolium counter ion, the lamellar mesoporous silica was synthesized [69].

### 3.4. SBA-15 Synthesis

The synthesis of the SBA-15 type of mesoporous silica involves the mechanism of co-operative self-assembly of the silica precursor around the template agent. The templating agent is typically the non-ionic poly(ethylene oxide)-block-poly(propylene oxide)-block-poly(ethylene oxide)-block copolymer, also known as Pluronic P123, comprising both hydrophilic and hydrophobic units, with larger molecules compared with CTAB [5]. The solubilization of the surfactant occurs owing to the association of the head groups of the hydrophilic poly(ethylene oxide) with water molecules through hydrogen bonds, and can be further improved in acidic media, as the hydrophilic groups will associate with hydronium ions [70]. The 2D hexagonal structure contains pores with a diameter varying between 6 and 10 nm, depending on the conditions of synthesis [5].

Thus, SBA-15 is usually synthesized through sol–gel methods, by solubilizing the Pluronic P123 surfactant in aqueous hydrochloric acid solutions and subsequently adding the silica precursor, such as TEOS or TMOS. The final structure is obtained by the calcination of the gel for removing the surfactant [71,72]. The SBA-15 mesoporous material is characterized by larger pores compared with MCM-41 and lower thermal, mechanical, and chemical resistance properties [27]. Additionally, to reduce the production costs for SBA-15 materials, other silica sources, such as bottom ash, could be used for the same synthesis route [73].

### 3.5. SBA-16 Synthesis

The synthesis of SBA-16 mesoporous material briefly involves the solubilization of block copolymers, such as Pluronic P123 or Pluronic F127, in aqueous hydrochloric acid solutions; the addition of silica precursors, such as TEOS; and calcination for surfactant removal [74,75]. Moreover, by modifying the silica sources, the stirring conditions, or the reaction temperature, a different architecture of the mesoporous materials could be obtained. Therefore, by adding inorganic salts such as potassium chloride as additives, the structural order degree can be increased and the micelles’ formation can be favored, by decreasing the concentration and the temperature of the critical micelle. Moreover, the pH value, the temperature, and the stirring conditions influence the hydrolysis degree of the silica precursor [76].

Similar to the SBA-15 materials, other silica sources, such as rice husk, could be used for synthesis in order to reduce the production costs [77]. Furthermore, other studies reported the use of micelle expanders—co-surfactants, including hexane, cyclohexane, 1,3,5-triisopropylbenzene, and 1,3,5-triethylbenzene—for obtaining SBA-16 materials with larger pore diameters [75].

### 3.6. FDU-12 Synthesis

The synthesis of FDU-12 mesoporous silica involves the use of the solubilization of the non-ionic block copolymer Pluronic F127 into a hydrochloric acid solution, the addition of the 1,3,5-trimethylbenzene/xylene/toluene and potassium chloride as additives, and TEOS as the silica precursor [39,78,79]. It has been proven that the temperature and duration of the synthesis reaction, as well as the concentration of the hydrochloric acid, silica precursors, and potassium chloride, directly influence the parameters of the MSN. Additionally, one group investigated the effect of the stirring rate, demonstrating that it can influence the growth of the silica mesophase and the structure evolution [78]. Moreover, the application of ultrasound conditions before the heating step has proven to generate MSN with hollow structures and more uniform size and shape [80].

### 3.7. Design Principle

As has been extensively exposed, the main constituents of MSN include the silica precursor, a surfactant (non-ionic or ionic) as a structure targeting agent, and a catalyst [15]. All these elements must be chosen in such a manner that their properties would confer all the desired characteristics to the intended MSN (Table 2).

The control of pore size is in strong accordance with the type of surfactant chosen, the use of co-surfactants, the precursor, as well as the conditions of synthesis. There is an addressed correlation between the molecular chain length and the pore diameter. As structure targeting agents, the molecular structure of surfactants is critical for determining the properties of the prepared mesoporous silica [81]. A surfactant molecule characterized by a high hydrophobic sequence will produce pores with larger diameters, which can be increased even further by the addition of co-surfactants, hydrophobic molecules that are able to accommodate within micelles, in closer contact with the hydrophobic moieties of the surfactants.

Aiming to elucidate the role of bile acids, Travaglini’s experiments demonstrated the influence on the shape of silica particles owing to the specific interactions between bile acids and CTAB. In this manner, bile acids and CTAB-composed cationic templating systems generate submicronic MCM-41 particles with different shapes, high porosity, and remarkable countenance. Furthermore, the bile acid is the element that allows the variation of particle architecture [82].

Han′s study reveals that the concentration of the templating agent is the main factor in controlling the morphology of the particles. On one hand, highly-organized mesoporous silica hollow tubes were produced by a sol–gel method with the ionic 1-decyl-3-methylimidazolium chloride and the non-ionic surfactant Pluronic P123 as the co-template and, on the other hand, using only 1-decyl-3-methylimidazolium chloride as the template, silica hollow spheres with disordered mesopores were obtained [83].

Thanks to its tunable structure, mesoporous silica plays an important role and is often chosen for the design of different types of multifunctional applications. Starting from the fluorescent properties of tetraphenylethene (TPE), luminogen-functionalized Gemini surfactant (C-TPE-C-6-C-TPE), which also associates with CTAB to regulate their construction in the structure-directing process, Yan offers important insights for the facile production of advanced fluorescent mesoporous silica materials [84]. Moreover, Li’s study also investigates the influence of the Gemini surfactant structure in the arrangement of MSN, especially regarding their morphologies and the molecular interactions that occur in the condensation of the silicate anions. Therefore, ordered mesoporous silica has been prepared by applying dehydroabietic acid as starting material and a series of rosin-based Gemini surfactants [81].

Furthermore, Morsi has reported the preparation of silica materials, such as MCM-41, SBA-3, SBA-15, silica nanoparticles, and silica microparticles using a simple, eco-friendly approach based on the sol–gel method. In this manner, promising silica materials with high surface area and mesoporous features have been produced [85].

Nevertheless, it has been shown that even an apparent problem such as the incomplete removal of the surfactant from the pores could be of interest because it provides basic catalytic activity to the as-synthesized MCM-41 silica, with important applicability in the transesterification reaction, achieving high conversions even under mild reaction environments [86]. However, it is highly recommended to ensure the structural stability of catalysts, especially when they are intended to be used in applications that require extreme reaction conditions or if they are used together with an aggressive solvent. In this context, just by including an additional hydrothermal treatment during the synthesis process, the stability of the materials could be considerably increased [86,87].

Hence, it can be stated that, even using the similar raw material, the characteristics of the obtained materials could be varied by changing different parameters, such as pH or the type of the surfactant template.

There are various patents regarding the synthesis of mesoporous silica particles, each describing the precise steps involved and their possible applications [88,89,90,91,92].

## 4. Surface Functionalization Strategies

As the main applications of mesoporous silica are based on its ability to effectively host within the pores great volumes of molecules of interest and to protect them against premature degradation, several surface functionalization strategies for enhancing its performances have been developed. Nonetheless, the attachment of specific functional agents onto the surface of MSN is fundamental as the remaining silanol functional groups have a tendency to interact with the surrounding molecules, causing uncontrollable release behaviors [43]. Moreover, surface modifications could further improve the biological responses through an increased biocompatibility and cytocompatibility, and reduce premature removal from the body by controlling the interactions with the cellular membrane compounds and plasma proteins [43,93,94].

The functionalization of MSN focuses on modifying the surface properties of the nanoparticles, such as the surface charge, or attaching functional groups or specific molecules that would further influence the interactions with the loaded active molecules, but also with the surrounding environments [93]. Additionally, intensive work has been performed in the development of stimuli-responsive systems, which could release the bioactive molecules as a response to the specific stimuli, such as temperature, pH, magnetic and electric fields, ultrasounds, light, redox agents, or enzymes [43].

**Figure 5:**
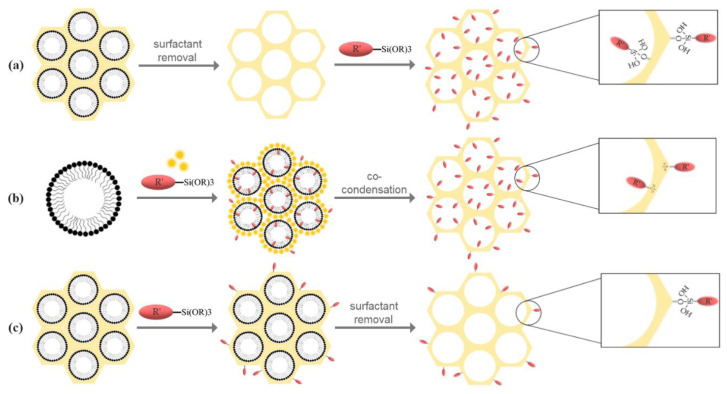
The main strategies for surface functionalization: (**a**) post-synthetic grafting; (**b**) co-condensation; (**c**) post-synthetic grafting followed by template removal.

In the case of MSN, silane chemistry is the main strategy for surface modifications [94], which further employs post-synthetic grafting, co-condensation, and post-synthetic grafting followed by template removal (Figure 5) [94,95], for enhanced blood circulation times, biological barrier permeation, and targeting delivery [14].

The post-synthesis functionalization can be achieved through physical impregnation or chemical grafting. The physical impregnation includes the dissolution of the compound containing the functional group, such as the amine, in a solvent and the addition of silica to the solution, followed by the evaporation of the solvent. The advantages of this method are represented by the simple and mild synthesis conditions. However, as the amine is physically adsorbed into the pores, the stability of the amine–silica composite represents a considerable limitation [23]. The process of chemical grafting is the most common strategy for the functionalization of mesoporous silica. This method involves the subsequent modification of both inner and outer surfaces of the MSN through the condensation of specific silane derivates containing the organic groups of interest (Figure 5a), including organosilanes [(RO)_3_SiR’], chlorosilanes [R_3_SiCl], or silazanes [(R_3_Si)_3_NH], where R’ is an organic group and R is an aliphatic radical, such as methyl or ethyl [94,96,97,98]. As the silica synthesis and the functional group incorporation steps are separate, the textural properties of the support can be independently controlled [23]. Furthermore, the post-synthesis functionalization of mesoporous silica can be achieved through direct grafting or primary modification, formerly described as secondary grafting, which implies the reaction between a previously grafted functionality with another functional group, and transformation of the grafted groups by additional treatments [99]. The yield of the post-synthetic grafting depends on the number, accessibility, and reactivity of the hydroxyl groups and on steric factors and diffusion of the molecules [23,98]. There are several advantages associated with the post-synthetic grafting method, such as enhanced retaining of the mesostructure and an enhanced control over the synthesis conditions [96,97,98]. Additionally, it allows for the attachment of macromolecules, such as polymers or lipids, to the silica walls. The strategies for polymer grafting include the “grafting onto” method, involving the prior synthesis of the macromolecules and the subsequent reaction with the silanol groups, and the “grafting from” method, where the polymers are grown from the MSN surface using specific precursors [94,97,100]. Moreover, through distinct bioconjugation methods, peptides, proteins, enzymes, antibodies, and aptamers can be attached on the silica surface [94]. However, the post-synthetic grafting might lead to a considerable decrease of the surface area, pore volume, and pore size. Nonetheless, the main disadvantage of this method is the non-homogenous distribution of the grafted groups within the surfaces, which can further lead to a preferential reaction at the pore openings, restricting or blocking the diffusion of the molecules to the center of the pores [96,97,98].

Additionally, post-synthetic modification can be assured by controlled chemical dissolution of the silica network; this can be easily done by treating the mesoporous silica (as well as many zeolites—microporous materials) with sodium hydroxide solution. In this case, silica is partially dissolved, and the pore size increases slightly, without a major modification of the surface hydrophilic/hydrophobic ratio [101].

In certain conditions, the synthesis of the functionalized mesoporous silica can be performed in a single reaction vessel. This route, known as co-condensation, direct synthesis, or one-pot synthesis, involves the simultaneous reaction of the alkoxysilane, usually TEOS or TMOS, and an organosilane, such as trialkoxy-organosilanes and chloro-organosilanes [94,102,103], in the presence of the surfactant template [97,99]. The process of co-condensation comprises the formation of a non-hydrolysable covalent Si–C bond between the organosilane and the siloxane species and the subsequent hydrolyzation of the siloxane species to form the silica network. As most organic functionalities are hydrophobic, they tend to align with the hydrophobic tail of the surfactant and, therefore, modify the inner and outer surfaces of the silica walls [94,99]. In this approach, the surfactant must be removed through solvent extraction, as calcination might destroy the organic groups due to high temperatures (Figure 5b) [97]. The content of silanol and functional groups can be tuned through the modulation of the molar ratio of the silica precursor and the organosilane, but alterations in the mesopore periodic ordering and particle size and shape must be considered [95,97,99]. The direct synthesis of functionalized mesoporous silica leads to a more homogenous distribution of the functional groups on the surfaces in a reduced number of synthesis steps and time and with minimal chemical waste [96,97,99,102,103], by circumventing several purification procedures [102]. Although the grafting method leads to more thermally stable networks with higher structural organizations, the co-condensation procedure can achieve enhanced surface coverage without alterations of the diffusion of molecules within the pores [103].

The route of post-synthetic grafting followed by the removal of the surfactant (Figure 5c) might be the preferred route if the functionalization must be performed solely on the outer surface of the MSN [95].

### Stimuli-Responsive Mesoporous Silica Systems

After endocytosis, nanocarriers are predisposed to cellular digestion through the mechanisms of endosomes and lysosomes. However, as it necessary for nanoparticles to release the therapeutic cargos into the cytoplasm or nucleus, subsequent modifications of the surface properties are fundamental. It has been proven that modified MSNs could escape the endo-lysosomal action and enter the cytosolic compartment, where they release the therapeutic cargo [104].

**Figure 6:**
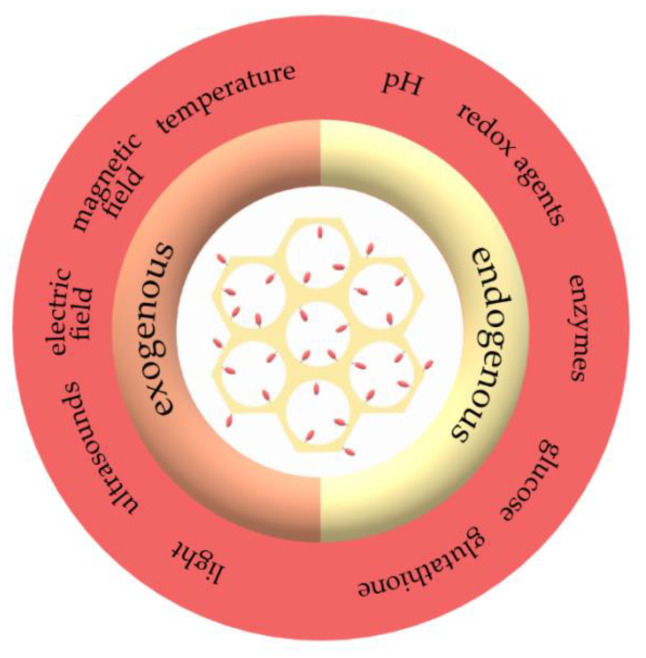
The main exogenous and endogenous stimuli involved in stimuli-responsive mesoporous silica systems.

Stimuli-responsive drug delivery systems offer a means to mediate the therapeutic cargo release under the action of specific triggers, such as extracorporeal physical or endogenous biological stimuli (Figure 6) [105]. This process is especially beneficial in cancer therapy, as tumor tissues exhibit common properties that differ from normal cells, including overgrown and permeable vasculatures, acidic and hypoxic environments, and overexpressed cancer-specific enzymes in the extracellular matrix [106]. Additionally, triggered drug delivery is important because of the very high toxicity these agents might cause to both normal and tumoral cells and tissues, and the systemic toxicity is usually avoided by local administration.

On one hand, exogenous stimuli include temperature changes, magnetic and electric fields, ultrasounds, and light, and they represent a promising solution to the heterogeneous physiological conditions of human population [107]. On the other hand, endogenous stimuli, such as pH, redox agents, enzymes, glucose, and glutathione, provide non-invasive and site-specific release of drugs in a spatiotemporally controlled manner. Therefore, these stimuli-responsive drug delivery systems can selectively deliver drugs into tumor tissues [105,107].

As the pores within the mesoporous silica are involved in the slow release under physiological conditions and cannot block the molecules to prevent the premature release, further modifications are necessary to enable the specific response to chemical and physical stimuli in diseased tissues [108,109,110]. There are several strategies to ensure the controlled release of the cargo from the MSN-based drug delivery systems, namely, particle surface coatings, pore sealing using molecular or particle gates, or cargo coupling to the walls of MSN. These approaches are further based on two response types, including the destruction or the conformational change of the pore sealing agent and the use of cleavable bonds that are broken upon exposure to specific stimuli [110].

Owing to its capacity to encapsulate large amounts of drug molecules into the pores, various options to seal the mesopores that reduce the premature release, and the stimuli-responsive release of the drug molecules, mesoporous silica is a versatile framework for designing stimuli-responsive drug delivery systems [106]. The variety of functional groups that can be attached on the silica walls, such as vinyl, acrylic, amines, epoxy, and thiols, offer the possibility for further attachment of stimuli-responsive macromolecules that improve the loading, targeting, and release of drugs [111]. Numerous studies have focused on the development of stimuli-responsive drug delivery systems. These systems can be categorized into single stimuli-responsive systems, based on the drug release under the action of a single signal and characterized by more facile synthesis and drug release mechanisms; dual stimuli-responsive, combining two different stimuli, such as pH/redox agents- and pH/temperature-based systems; and multiple stimuli-responsive systems, that will release the cargo as a response to three or more stimuli, with fewer reports owing to the increased number of steps and complex principles involved in the synthesis and release of such systems [112].

## 5. Application of Mesoporous Silica Nanoparticles

### 5.1. Biomedical Applications

As is widely known, cancer is one of the deadliest diseases that affects many people around the globe and, therefore, it has attracted considerable scientific attention. The most common treatments for cancer are surgery, radiotherapy, and chemotherapy, but most patients are affected by the associated severe side effects. Hence, scientists are trying to develop new modalities by engineering specific drug delivery systems that target tumor tissues more precisely through the help of smart nanomaterials and nanoparticles [113,114]. The tendency nowadays is to enlarge the use of nanotechnology by binding together various sciences of engineering and biomedicine, with the main purpose of diagnosing and treating cancer.

Nanotechnology has a wide range of utilities in cancer treatment, which offer trustful effective responses to the conventional diseases. To be able to reach the cancerous cells, it is necessary to design a nanobiomaterial that can entrap therapeutic agents. For drug delivery applications in cancer treatment, numerous organic and inorganic nanocarriers, such as liposomes, protein nanoparticles, polymeric nanoparticles, quantum dots, and ferro-fluids, have been studied. Moreover, diverse types of mesoporous materials were investigated as mesoporous carriers, including carbon, alumina, silica, titanium, iron oxide, and niobium oxide, but their biomedical applications were restricted owing to their limited biocompatibility. Some of them were already used as drug delivery systems, for example, mesoporous alumina with pores ranging from 2 to 7 nm was used as a model drug ibuprofen and mesoporous carbon nanoparticles encapsulating doxorubicin agent was investigated for chemo-photothermal therapy in breast cancer [110,115,116,117,118,119].

Nanomedicine is the link between nanotechnology and biomedicine having various applications in disease treatment, especially diabetes, tissue engineering, and cancer theranostics. Theranostic nanomedicine is rising as an important positive feature in using nano-platforms for both imaging and therapeutic applications. More precisely, “theranostic” means that all achievements from medical and pharmaceutical fields focus on developing therapies and integrating diagnostic and therapeutic features into a single substitute. MSN, iron oxide nanoparticles, quantum dots, carbon nanotubes, and gold nanoparticles were used as potential nano-platforms for theranostic nanoparticles, but MSN was the most extensively researched material having interesting applications in biomedicine and nano-therapeutics [115,116,120,121,122].

Nanoparticles applications have completely changed the understanding and treatment of various diseases. As already presented, nanopharmaceuticals have a major impact on reducing the side effects and enhancing medical care. The fact that MSNs could connect various types of therapies is favorable because, through functionalization, they could load on their pores different drugs in order to make the final agent the perfect asset to treat and diagnose complicated tumors. Moreover, diverse compounds such as molecules, ions, cellular materials, and contrast agents could be incorporated, which have applicability in bioimaging assessments. Nanomedicine could bring a great impact on human health, but in order to help the world, the barricade between clinical tests and commercialization must be overcome. As already mentioned, MSNs are perceived as highly impressive “nanomedicines” in both therapeutic and diagnostic characteristics. The Food and Drug Administration (FDA) considered and approved the introduction of silica in pills manufacturing and nutritive agents for hair, nails, and skin. An important perspective for MSN should also be focused on manufacturing effective new controlled drug release adequate in targeting specific tumor cells and developing proper diagnosis and therapy treatments [41].

The first drug delivery system used in biomedicine in the 1960s was liposomes, with current strategies also focusing on polymers, silica, metal oxides, and semiconductor nanocrystals. The first nanodrug approved by the United States Food and Drug Administration (FDA) is Doxil, which is doxorubicin encapsulated in liposomes for minimizing the side effects in heart tissue muscles. However, the most studied and auspicious nanomaterial used for tumor drug delivery systems is MSN [115]. The first silica nanoparticles for tumor diagnosis were Cornell dots (C-dots) approved by the FDA for the first stage human clinical trial [114]. The most highly appreciated system is mesoporous silica, which gained important consideration in biomedical applications because of its tunable pore size, high surface area, surface functionalization, chemical stability, and porous nature, which allows for a high loading output. The porous nature helps release the adsorbed drug molecules into the tissue, which makes mesoporous silica a considerable candidate as a drug delivery carrier. However, mesoporous silica has to be target-specific and to release the drug on the specific target site. To overcome this issue, the pores of mesoporous silica could be opened by internal or external triggers or stimuli like electromagnetic radiation, magnetic and electric fields, ultrasound, and different chemical species. In this context, its external surface could be tuned with biochemical or supramolecular structures, which act as molecular gates, being able to control the diffusion of loaded drug inside the mesoporous channels under the action of an external stimulus. Furthermore, different types of external stimuli were investigated, but the chemo-responsive system provides more effectiveness when it comes to reducing adverse effects. This chemo-responsive effect is acquired by attaching a certain gate-keeper molecule on the pore that adjusts the drug release [110,115,116,117,118,119,123].

**Figure 7:**
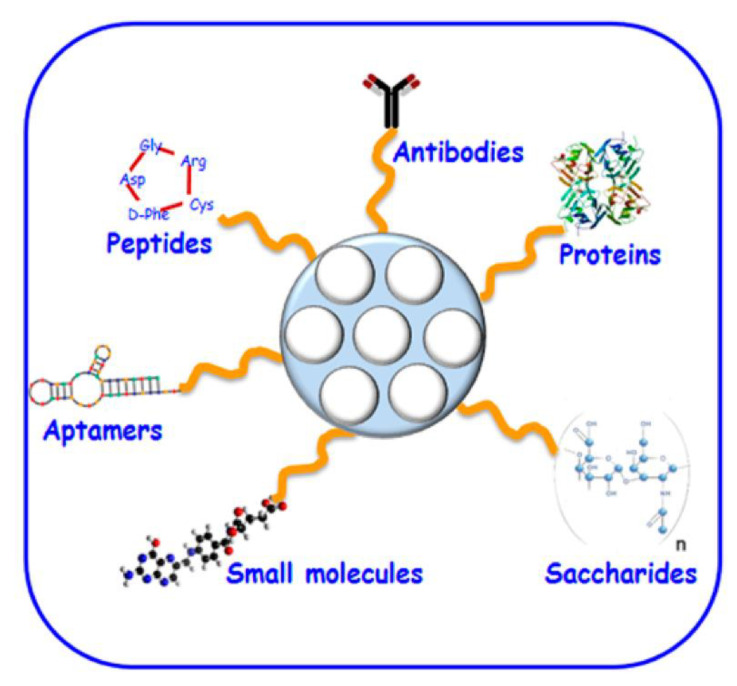
The possibilities of attaching (macro)molecules on the surface of MSN for active targeting. Reprinted from an open access source [16].

Moreover, MSNs were considered suitable candidates owing to their pore and particle size, which can be adapted in a wide range, for example, from 2 to 50 nm and from 10 nm to microsized particles, which will allow for a more facile management of the “resident” molecules. Another special feature of MSN is the capability to conjugate metals and metal oxides, different ligands, polymers, and fluorescent agents through the reactive silanol groups. Owing to this interesting property, MSN could bring a great contribution in tumor targeting, bioimaging, and stimuli-responsive release. Additionally, MSN could be used in drug delivery for loading biomacromolecules such as proteins that could be encapsulated in the silica nanoparticles or adsorbed onto the surface (Figure 7) [124,125].

An interesting approach of mesoporous silica was to use its surface to activate drug release. This phenomenon occurs when different molecules are entrapped on their pores, which further controls the drug release into the system. The controllable release of the drug takes place only under the action of internal or external stimuli, which have unique responsiveness. The response obtained using chemical species (ions or molecules), termed as chemo-response, allows for the release of the drug at the specific tumor site with no adverse effects [110]. Another interesting characteristic of MSNs is their “zero premature controlled release”, which involves embedding drugs inside and “luring” other materials on the surface to obstruct the pores. The most important challenge is to bind the bridge between in vitro and in vivo experiments, so by multi-functionalizing the surface, MSNs were able to target cancer tissues and release the drug load into the specific area [114].

Biocompatibility is an important issue that must be taken into consideration when we think about studying the toxicity of MSN [115]. In order to study their toxicity, their size, shape, and surface may bring new understanding regarding their biocompatibility. An interesting fact that needs to be mentioned is that some nanoparticles, including MSN, can be encapsulated in tumors through passive absorption owing to the enhanced permeability and retention (EPR) effect. Silica is already recognized as safe by the FDA and is already used as an excipient in drug tablet formulations. As MSN toxicity and biocompatibility highly depend on their properties, more studies are still necessary for developing standardized assessments [116,117,126]. Numerous reports have confirmed that, depending on the synthesis approach, silica nanoparticles have low cytotoxicity. Thus, the aim is to use the final product in medical trials and actually apply MSN in tumor-targeted delivery systems [113,114].

MSN applications in nanomedicine are related to therapeutics, diagnostics, and theranostic treatments. Firstly, MSNs are generally applied for therapeutics owing to the fact that they can adjust their surface in order to deliver drug molecules to cells and tissues, and they can also behave as gene carriers for DNA and RNA. In molecular therapy, MSNs help identify the proper cancer treatment. The main target in cancer therapy is focused on finding new strategies to beat cancer and, at the same time, reducing the side effects of the drugs. The most studied non-cancerous approaches have applicability in therapeutic applications of many diseases like diabetes, rheumatoid arthritis, and HIV. Secondly, MSNs are being used for diagnostics in the field of molecular imaging and position emission tomography (PET) for diagnosing and monitoring the therapeutic strategy through noninvasive techniques. MSNs are also intensively used in different bioimaging procedures, which include computed tomography (CT), MRI, optical imaging, and ultrasound [93,115,121,122]. MSNs have been widely used to diagnose cancer by providing a great cargo for contrast agents in MRI. Various studies aimed to functionalize methionine-MSN with gadolinium ions in order to obtain selectivity and non-toxicity. For instance, the cells charged with gadolinium ions-MSN exhibited dark signals in the in vivo MRI analysis, but there are some formulations where gadolinium charged with methionine-functionalized MSN presented toxicity on MCF-7 breast cancer cells. Different studies illustrated lanthanide nanoparticles covered with MSN for stimuli-responsive release of doxorubicin under MRI technique specialized in cancer treatment [14,122]. Thirdly, theranostics involves manufacturing or designing a platform with multiple purposes, with both diagnostic and therapeutic properties [93,115,118,122].

Besides pore dimension, whose influence on different mesoporous materials properties was intensively studied, other morphological characteristics, such as shape, have been brought under the attention of researchers. Shao et al. have comparatively studied the effects of varying the shape and aspect ratio of magnetic MSN on the endocytosis, biocompatibility, and biodistribution, which could provide the means for considerably improving the efficiency and safety of MSN systems in cancer theranostics. Their silica-coated superparamagnetic nanoparticles conjugated with fluorescent, as well as targeting moieties, showed a higher cellular internalization of rod-like nanoparticles compared with sphere-like nanoparticles [127]. Similarly, the effect of shape was the subject of Zhang’s study, who synthesized two types of MSNs in order to investigate an optimal incorporation of indomethacin within the mesopores. The results of the study proved that the mesoporous silica with helical channels and a rod-like shape exhibited better features for developing drug delivery systems [128]. A more elaborate method of drug photo-release is described by Hernandez and involves laser irradiation of gold nanostars (AuNSts) coated with a mesoporous silica shell and capped with paraffin. The AuNSt compound has the particularity of increasing its temperature by hundreds of degrees (°C) under a laser wave, even at low wavelengths. Doxorubicin-loaded nanoparticles showed no cytotoxicity toward HeLa cells, until they were irradiated with an 808 nm laser, leading to paraffin melting and drug release [129].

In order to avoid premature cargo leakage and uncontrolled release, a problem that often occurs when an MSN drug delivery system is designed, Adhikari et al. developed an approach for modifying bare MSN to inhibit burst cargo release. He proved that the metal organic framework (MOF)-functionalized MSN containing doxorubicin is superior to the bare MSN-based drug delivery system. The design of the system involved the encapsulation of doxorubicin inside MSN and the subsequent formation of composites through an in situ room temperature reaction in the aqueous medium with two biocompatible MOFs, namely iron 1,3,5-benzenetricarboxylate and zinc 1,3,5-benzenetricarboxylate. Cytotoxicity tests on HeLa and mouse embryonic fibroblast (NIH3T3) cell lines proved the biocompatibility of the composite systems and cellular uptake tests revealed the incorporation within the cells without any changes in terms of morphology [130].

Having the great advantage conferred by its tunability, mesoporous silica is also suitable to be used in multifunctional nanomagnets, such as those proposed by Li et al. A nanocarrier based on mesoporous silica-coated iron oxide nanoparticles comprising the anti-cancer drug doxorubicin encapsulated within the pores and the matrix metalloproteinase-2 enzyme responsive peptide covalently linked onto the surface of the nanoparticles [131].

Cancer therapy research has attracted intensive attention and scientists have designed an attractive core-shell nanocarrier with tree coatings, each of them being receptive to a specific stimulus, and the core system containing MSN. The first shell consists of a fluorescein isothiocyanate-hyaluronan layer, which has an enzymatic response; the second layer, in disulfide-SiO_2_, for glutathione responses; and the third shell, represented by the zwitterionic exterior, which is sensitive to certain pH levels. Owing to increased absorption and permeability properties of the nanocarriers around tumor cells, the zwitterionic exterior is being influenced by the pH and becomes positive, which occurs in the benefit of cellular recovery, as long as the negative nanocarrier interconnects with the positive cellular membrane. Once incorporated into the tumor cells, the large amount of glutathione absorbed in the cytoplasm could be attached to disulfide in order to eliminate SiO_2_ and the hyaluronan would be disclosed, which will activate the drug release. The purpose of the research was to manufacture a drug delivery system coated with different materials having diverse functions, which would promote extended blood circulation times, effective tumor absorption, and drug release stimulated through different stimuli. More precisely, the core of MSN acts as a capsule for anti-tumor drugs, while the hyaluronan and disulfide bonds modulate the release of the drug through the deterioration owing to high amounts of hyaluronidase and glutathione in tumor cells. Moreover, the addition of fluorescein isothiocyanate allowed for drug tracking and tumor cell imaging [132]. Furthermore, MSN-hydroxyapatite nanocarriers have good drug loading and biocompatibility and great anti-tumor effect, which may affirm that silica-based nanoparticles could enlarge the understanding of designing safe bio-drug systems [113,122].

After thorough studies, it was stated that MSN presented interesting characteristics with applicability in drug/gene delivery systems and, especially for cancer therapy, they have shown noticeable advantages, such as exemplary drug loading cargo and endocytosis nature. In this regard, the external surface of MSN can be enhanced with different memory-tumor molecules and stimuli in order to obtain better therapeutic anti-cancer effects. For instance, in gene delivery, the porous surface of the MSN can encapsulate and preserve different genes, being an exceptional agent in drug/gene delivery (Figure 7). Even though there is a great number of studies regarding the advanced progress of MSN cargo for drug/gene delivery, there are important arguments to consider their improvement, and the ones that are worth mentioning and have been intensively investigated are biocompatibility, degradability, and pharmacokinetics [42,125].

Breast cancer is considered a deadly disease, with the unfortunate prognostic that one-in-eight women will develop breast cancer in their lifetime [14]. Researchers are focusing their efforts on developing new approaches using the biotechnology of MSNs in breast cancer therapy to create multiple platforms to treat and diagnose. Although there are numerous in vitro and in vivo applications, researchers are investigating new cancer biomarkers in order to detect and determine a precise and correct diagnostic for each patient. An important issue is related with the cooperation between researchers and pharmaceutical industries, which would speed up the decision process of these conceptions [14,118].

MSNs can also be successfully applied for the development of antimicrobial platforms. Studying the encapsulation of the antibiotic clofazimine within the pores of mesoporous silica particles with hydrophilic and hydrophobic surfaces, Angiolini et al. demonstrated that modifying the hydrophilicity/hydrophobicity of mesoporous silica particles could represent a promising strategy to control the release of the drug without affecting their loading capacity. Additionally, the fluorescent images revealed an optical “halo effect” that could be interpreted in regard to specific quenching of high concentrations of molecules [133].

Besides cancer, diabetes will affect almost 333 million people by 2025, which means that 90% of these people will have type 2 diabetes. Diabetes is a metabolic disorder that appears owing to a reduction of insulin and, if not treated in time, it could have severe repercussions on the nervous system, heart, kidneys, eyes, and skin. Trying to solve the problems associated with diabetes, different delivery systems using mesoporous silica as a substrate for the controlled release of insulin into the stream were developed. MSNs are considered important “tools” in normalizing the insulin delivery and, because of this, they can be decorated with different compounds in applications as biosensors for glucose monitoring [116,117,134].

Another important application of silica-based mesoporous nanobiomaterials is in bone tissue engineering, which could have a significant impact on human health. A large percentage of the human population have bone tissue disorders and there is an increasing need for a modality to treat these conditions. However, the most common treatment is to remodel the bone under surgery by implanting a suitable natural or synthetic material. The implantable material must be biocompatible, osteoinductive, and osteoconductive and it must be osteointegrated within the defected natural bone tissue, where it should promote the formation of a new bone tissue using cells, extracellular matrix, intracellular communications, cell–matrix interactions, and growth factors. Silica-based mesoporous nanobiomaterials have been shown to be extraordinary nanocarriers for the delivery of drugs and biomolecules both in vitro and in vivo. Furthermore, MSNs have been widely applied as polymer reinforcement within acrylic in bone implants [135]. An innovative composite material consisting of mesoporous silica and hydroxyapatite has been evaluated for its potential as drug nanocarrier and filling material in a polymer matrix in order to design poly(lactide-co-glycolide)/mesoporous SiO_2_-hydroxyapatite composite material, a bone tissue scaffold with drug release capacity [136]. The natural bone is highly complex and the link between growth factors and bone regeneration must be found. Thus, scientists are still trying to develop bone tissue engineering techniques to overcome the challenges associated with conventional treatment methods [135].

### 5.2. Cosmetics

**Figure 8:**
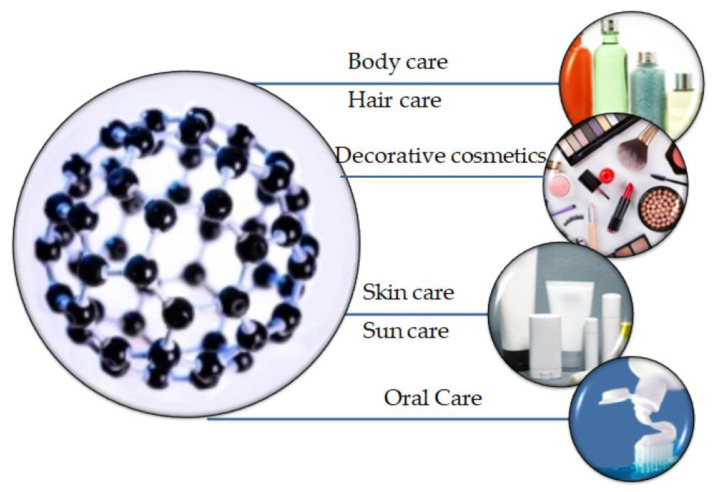
The applications of nanomaterials in the cosmetic field. Reprinted from an open access source [139].

Besides its applicability in the biomedical field, nanotechnology, through the production of nanoparticles, has been recognized for its efficiency in the cosmetic area. Specifically, their application is highly advantageous owing to the possibility of controlling the release of the active substance within the cosmetic formulation and the increased effectiveness, stability, and safety they provide [137]. In this regard, nanocosmeceuticals have received considerable interest in the last years owing to their wide use in skin, hair, and nail products for preventing wrinkles and photoaging, hyperpigmentation, or dandruff and hair damage (Figure 8). However, the concern regarding the nanotoxicology of such nanocarriers is proportionally increasing owing to potential hazards they could cause, as nanoparticles are capable of permeating the skin [138].

Thanks to their hydrophilic surface, which allows for a protracted circulation and cost efficiency in regard to their manufacture, silica materials have been widely applied in the cosmetic industry [139,140]. Additionally, silica nanoparticles have shown to improve the efficiency, texture, and shelf-life of cosmetic products, as they possess high absorbance properties and act as anti-caking agents [139].

Specifically, silica nanoparticles have been widely used in lipstick formulation as they improve pigment homogeneity and distribution, thus preventing their migration or bleeding into the fine line of the lips [141,142]. In this context, there is a patent regarding the development of delivery systems comprising lipid nanoparticles that could be adsorbed onto the surface of MSN and further used for such types of applications [143]. Moreover, thanks to their adsorbance and slow release capacities, MSNs are generally added to leave-on and rinse-off cosmetic products [144].

Moreover, the MCM-41 platform has been developed as a nanocarrier for octyl methoxycinnamate. Although it has been extensively used as a UV filter with promising absorbing properties for sunscreen applications, octyl methoxycinnamate is characterized by photoinstability and possible skin permeation. However, the proposed system is promising for stabilizing UV filters within sunscreens owing to the presence of the mesoporous MCM-41 [145,146].

However, there are still some concerns regarding the use of MSN in the cosmetic industry, as size and surface modifications, owing to their high reactivity, might lead to increased toxicity of the products [147,148]. In this context, one study revealed that MSNs with particle size in the range of 1 to 5 nm are more toxic when compared with the equivalent dose of 10 nm MSNs [142]. Therefore, long-term exposure tests are fundamental for the safe application of MSNs in cosmetic products [139].

### 5.3. Fluorescent Sensing

The detection of chemical residues can be performed through fluorescent sensing, a promising tool that uses specifically designed fluorophores with aggregation induced emission (AIE) effects. This particularity is of great technological importance and it is represented by an emission upon molecular aggregation provided by its restriction of intramolecular motion (RIM) process [149]. AIE fluorophores have generally been used as chemical sensors for assessing the pH, explosives, carbon dioxide, amines, and metal ions as they are highly advantageous in terms of facile fabrication, ready functionalization, excellent stability, high signal-to-noise ratio, and low detection limit [149].

In this context, AIE luminogens-/AIEgens-functionalized MSNs were synthesized by Wang et al. through post-grafting two types of tetraphenylethene derivatives on the surface of the mesoporous materials. Under UV irradiation, these chemical sensors emitted strong blue fluorescence and exhibited fast and sensitive responses to 2,4,6-trinitrophenol and 2,4-dinitrophenol through the fluorescence quenching process [150]. Moreover, a system based on functionalized magnetite nanoparticles and MSN, using 2-(2-hydroxyphenyl) benzothiazole as the fluorogenic agent for the selective detection of zinc ion in live cell imaging, was reported by Erami et al. It was proven that the presence of the fluorophore in the composite system increased cell viability and zinc ion uptake, with desirable signaling in the normal human kidney epithelial (Hek293) cell line [151].

In another area of applicability, Liu et al. proposed a smart luminogenic-functionalized SBA-15 developed by incorporating tris(4-bromophenyl) amine into the mesoporous material. Tests revealed that this complex presents an improved emission based on “fixation-induced emission” strategy and could be used as a fluorescent sensor for the detection of antibiotics (e.g., cefalexin) in water [149]. Similarly, Deghgani et al. designed an efficient aptasensor for detecting kanamycin comprising the aptamer/complementary strand (dsDNA)-capped MSN and Rhodamine B as a fluorescent probe. The MSN pores were filled with Rhodamine B and subsequently capped with dsDNA. The presence of kanamycin resulted in the separation of the aptamer sequence from its complementary strand, which consequently uncovered the pores and led to the release of Rhodamine B. Therefore, the fluorescence intensity increased significantly, and the relative fluorescence intensity exhibited a linearity range between 24.75 nM and 137.15 nM of kanamycin and a detection limit of 7.5 nM [152].

Wang et al. offered a facile strategy for the fabrication of fluorescent MSN embedding various photothermal therapeutic agents, which provides a switchable platform for biomedical fluorescent imaging and a potential therapeutic system for cancer and other diseases. Specifically, a stable mesoporous fluorescent silica nanoparticle with integrated PEGylated MoS2 was synthesized and the targeted fluorescent bioimaging combined with stable photothermal effects against MDA-MB-23 cancer cells was demonstrated [153].

Moreover, speculating its sensitivity-colorimetrical properties, Li et al. created a silica–dye for the sensitive gas detection of cyclohexanone, a volatile marker of the 1,3,5-trinitro-1,3,5-triazinane and 1,3,5,7-tetranitro-1,3,5,7-tetrazocane explosives. The synthesis of the silica–dye composites included the hydrolysis of ultrasonically sprayed organosiloxanes under mild heating conditions, leading to the formation of micro-spherical, nanoporous structures with a high surface area (~300 m^2^/g) for gas exposure [154].

### 5.4. Bio Catalysis Involvement and Natural Processes Behavior

There has been a considerable scientific interest towards the development of nanostructured catalysts that mimic natural enzyme behavior without their inherent limitations, such as low stability, problematic biocatalytic performance in harsh media, and high preparation costs. In their large area of applicability, synthetic mesoporous silica materials are frequently used as supports for enzyme immobilization owing to their morphology, which allows for a high loading capacity, and exhibit proper mass transport capacity owing to its interconnected channels of mesopores. As presented in a recent study, these materials are also available from natural sources, an example being the biosynthesis capacity of the diatom class, which is a large group of microalgae. Physiologically, the diatom cells are contained within a unique silica cell wall known as a frustule made up of two valves called thecae that typically overlap one another [155].

In this context, there is a consistent correlation between the mesopores low volume fraction and the high enzyme activity preservation within the synthetic silica. The use of metal-containing MSN as the oxidase has been investigated by Aghayan et al. The study revealed that Fe-MSN holds great potential for the simple manufacture of low-cost biosensors that could be used for determining biologically important analytes, such as glucose and hydrogen peroxide [156].

### 5.5. Environmental Applications

The use of mesoporous materials for the removal or even degradation of several pollutants is considerably important. Worldwide, antibiotic resistance has become a major concern. The discharge of wastewaters (especially from hospitals, drug factories, chicken farms) generally determines a sub-therapeutic level and some microorganisms can develop resistance towards antibiotics. In this context, the development of adsorbents or materials which are able to degrade antibiotics, and not only, is mandatory.

Mesoporous materials, especially MSN, hold properties that make them excellent candidates in environmental applications [157]. Specifically, their high surface area and the presence of silanol groups on their surface allows for the adjustment of multiple properties, depending on the purpose of the application [158]. On the basis of the knowledge about mesoporous silica regarding their unique properties, such as non-toxic character, large surface area and pore volume, and tunable pore size and surface properties, MSN could be applied for the adsorption of pesticide and antibiotic residues that have unintentionally entered the environment [121,159,160].

In the past decades, pesticides pollution has threatened environmental and human health. One example of aggressive pesticides that affect the ecological environmental are organophosphorus pesticides, which have carcinogenic, teratogenic, and mutagenic repercussions on animals and humans. The improper application of organophosphorus pesticides in agriculture, which leads to high amounts of pesticides residues also recorded in fruits and vegetables, needs to be carefully controlled [161,162,163]. In this regard, researchers are making many attempts to manufacture new materials for monitoring the pollution with organophosphorus pesticides. Many new outstanding materials were developed, such as nanorods, nanoparticles, carbon nanotubes, and nanocapsules. Among them, the most suitable material with high absorption features was the core-shell magnetic materials impregnated with polymers. The most important aspect is to synthesize an effective and cost-efficient sorbent to trap these organophosphorus pesticides. However, it is necessary to establish the connection between the structure and the porous character of the sorbent [163,164,165].

From the wide range of sorbents developed to efficiently absorb the organophosphorus pesticides from the environment, MCM-22 was able to trap almost 100% of carbendazim, but its high cost blocked its application. In consequence, the idea was to fabricate new sorbents to entrap organophosphorus pesticides and to protect the environment. In conclusion, the SBA-15 and MCM-41 samples exhibited the higher absorbent capacity for carbendazim, while the in situ carbonized SBA-15 manifested high activity in the adsorption of phoxim, chlorpyrifos, and carbendazim. Fortunately, there was also an alternative sorbent, namely the silica coated with MgO, that was able to successfully remove folimat and rogor from water and has the advantage of being cost-efficient and simple to produce [163].

Furthermore, MCM-41 was prepared through the sol–gel method and encapsulated in a biomimetic polymer, polydopamine (PDA), which can attain a varied range of materials, such as ceramics, copolymers, and semiconductors, through oxidative polymerization. The purpose of this study was to use PDA coatings on the surface of mesoporous silica, as it is an excellent “trapper”, with the possibility to block different molecules and release them at lower pH values [159,166,167]. Accordingly, it was proposed to decorate PDA with triazolone/MCM-41 and, in order to ensure a sustained release and absorption properties, the system was coordinated with different metal such as Fe, Cu, and Zn. Many tests were carried out to compare different compositions of MCM-41, PDA-MCM-41, Zn-PDA-MCM-41, Cu-PDA-MCM-41, and Fe-PDA-MCM-41. Scanning electron microscopy (SEM) and TEM images revealed that the surface of MCM-41 was irregular and the PDA encapsulation led to the formation of a thin layer on the surface of the samples. Regarding MCM-41 adsorption capacity, it increased significantly after the encapsulation within PDA. Moreover, Fe and Zn ions improved the adsorption performance of the samples, up to 173 mg g^−1^ for the Fe-PDA-MCM-41, which was 160% more than that of MCM-41, while the Cu-PDA-MCM-41 decreased owing to the strong interaction between Cu ions and triazolone. When it comes to the release rate of the molecules under different pH values, the results varied. In acidic media, the lower the pH, the faster the release rate for the encapsulated sample with PDA, as the PDA coating was unstable under acid conditions with a tendency to hydrolyze. In alkaline media, the release rate decreased owing to the basic effect of the PDA coating. In conclusion, the as-synthesized system encapsulated by PDA exhibited important pH-sensitivity according to the stability of the PDA layer. Moreover, this system is expected to have great potential in applications such as manageable pesticide delivery under different pH release behaviors [159].

In order to evaluate the risk of exposing organisms to different chemicals, a multiple component mixture was developed to evaluate and predict their toxicity. To assess their dates, two models were used: the concentration addition model and the independent action model, with the precise purpose of evaluating the combined toxicity of the mixture [168,169,170]. As the evaluation of the combined toxicity of different mixtures with many mixture ratios and concentration levels is very difficult, an optimal experimental design was developed. As optimal experimental design studies are generally not comprehensive in a multiple component mixture system with more than three components, the equivalent effect ratio design or the fixed-ratio ray design were used. However, a mixture system with a precise chemical composition comprises a variety of different mixture rays with different concentration levels, and all the concentrations of the mixtures designed by the two methods are located on one ray in the concentration. Therefore, the prediction of the mixture toxicity or the representation of the whole mixture system are still not possible [171,172,173]. In this case, it is necessary to use an optimal experimental design model. These systems and experimental models were designed in order to test and predict the toxicity of different chemicals on a wide range of organisms. Nitrobenzene derivatives are a class of toxic chemicals extensively spread throughout the environment as they are intermediary products resulting from the production of explosives, drugs, dyes, fungicides, and other industrial compounds. Although they are prohibited in many countries owing to the toxicity they pose to living organisms, they are still presented in the environment. The toxicity of five nitrobenzene derivatives against various aquatic bacterial strains, such as *Scenedesmus obliquus*, *Daphnia magna*, and *Vibrio qinghaiensis*, was intensively studied, demonstrating that the toxicity of any mixture within the system is predictable regardless of the concentration ratio or level [172,173,174].

The extensive presence of hydroxyl radicals, first discovered in 1934 by Haber and Weiss during the Fenton reaction, in different media, including natural waters, atmosphere, biological systems, or interstellar space, is well known. Hydroxyl radicals are highly reactive and predisposed to steal hydrogen atoms from other molecules in order to form water molecules as they form through the bonding between a hydrogen atom and an oxygen atom. Their reactivity in association with other various water pollutants, including bacteria and organic or inorganic compounds, is intensively researched, as it is the main focus of the agency pollution in wastewater treatment management. In this context, it is necessary to remove or transform hazardous organic and inorganic pollutants present in waters and wastewaters into secondary compounds that are less toxic. Thus, advanced oxidation processes are mechanisms with this capability to remove or transform pollutants in less toxic materials. Advanced oxidation processes use highly reactive hydroxyl radicals as primary oxidants. In advanced oxidation processes, hydroxyl radicals are usually generated by coupled chemical and/or physical systems that consist of H_2_O_2_/Fe^II^ or H_2_O_2_/Fe^III^ (Fenton), H_2_O_2_/catalyst or peroxide/catalyst (Fenton-like), O_3_ (ozonation), and H_2_O_2_/O_3_ (peroxone), which is generally associated with an irradiation technique, namely vacuum-UV radiation, UV radiation, and ultrasound. Owing to their high oxidative capacity, hydroxyl radicals are known as the “detergent of the atmosphere” [175].

Another important study regarding the degradation of pesticide involves the carboxylesterase immobilization from *Spodoptera Litura* in mesoporous molecular sieves. MCM-41 and SBA-15 were used for the study as mesoporous sieves, namely as enzyme templates for comparing the efficiency of immobilization. At this moment, there are various innovative methods applied to the degradation of pesticides, such as the photocatalytic and enzymatic degradation [176,177,178]. Besides these methods, the enzymatic degradation and hydrolysis displays promising applications owing to its high biocatalytic and environmental features. Owing to the presence of hydrolases groups, carboxylesterase exhibits the capability of hydrolyzing carboxyl esters. For instance, the esterases from insects have a primordial role in the degradation of organophosphates, carbamates, and synthetic pyrethroids insecticides. Thanks to their ability to hydrolyze these insecticides, carboxylesterases are very important in developing products that deal with insecticides pollution. After studying different materials, it was established that enzymes immobilized on mesoporous supports show better thermal and pH stability than free enzymes. As a conclusion of this study, it has been reported that carboxylesterase immobilization in SBA-15 was a better support in the investigation regarding the stability of the immobilized enzyme [178,179].

γ-hexachlorocyclohexane is a chlorinated pesticide lindane used in wood treatment and preservatives for new timbers in the United Kingdom and for the destruction of wood boring insects. Lindane is an accumulative, long-lasting toxin known to damage the liver and to promote cancer and caused by the slow release over long period of time [180]. Researchers focused their attention on solving the problem of pesticide emissions in the environment by applying a zeolite coating to pesticide-treated timber, which revealed that the zeolite can effectively reduce lindane emissions, even in the presence of reagents, such as toluene, and moisture from the air [181]. The best sieve chosen for the selective retention of lindane emissions from treated timbers was zeolite molecular, which has a polar nature, and the uniform pore size of the zeolites would enhance the selective uptake of lindane in advantage to toluene. In the study, activated NaY zeolite with no water (dry) in its pores and non-activated (wet) NaY zeolite containing preabsorbed water were compared in order to assess their ability to adsorb lindane and toluene in both the gaseous and liquid environment. With the purpose of minimizing the water sorption, a silanised NaY zeolite with a hydrophobic coating was introduced in the study. An MCM-41 aluminosilicate zeotype material was also introduced in the study, chosen because the pore size of the MCM-41 was very similar to the system in the zeolite Y. The conclusion of the study was that, in the liquid phase, activated NaY zeolite is selective for lindane in advantage to toluene at all concentrations, but the selectiveness for lindane on wet NaY depends on the concentration. Gas chromatographic tests demonstrated that NaY detained both lindane and toluene for more than 120 min at 200 °C, in contrast to the MCM-41, which resulted in 20 min for lindane and less than 1 min for toluene. Additionally, the thermal gravimetric analysis suggested that the uptake of lindane and toluene in gas phase for both activated and wet NaY zeolite was from 240 to 260 mg g^−1^. The silanized NaY zeolite increased the uptake, while MCM-41 also increased the uptake of 100–150 mg g^−1^ for all activated, wet, or silanized materials. Hence, it was proved that lindane can remove water and toluene from within the pores of NaY and MCM-41 in both liquid and gaseous phases. In this manner, a promising approach for producing coating materials for reducing pesticide emission from treated timbers to the environment was developed [181].

Ever since their discovery in 1928 and their widespread use since the 1940s [182], antibiotics have been used as the first-line treatment for bacterial or protozoan infection, for preventing infections after surgical wounds or dental procedures, or for immunomodulation [183]. Thus, antibiotics are chemical substances with the capacity to inhibit the growth or kill microorganisms by interfering with their metabolic processes [184,185]. In this regard, MSN can be designed as carriers for the delivery of antibiotics, as they could retain them inside their mesopores. An example for such a type of applications is represented by the synthesis of drug delivery systems consisting of gadolinium nanoparticles with a paramagnetic nucleus and a mesoporous silica shell designed for the release of either the chlortetracycline antibiotic, or the anticancer drug chlorambucil. The well-defined pores and their hydrophobicity allowed for the successful immobilization of these water-insoluble drugs [186]. Furthermore, such systems can be introduced in hydrogels for wound healing purposes. In this regard, one study reported the development of MCM-41 nanoparticles loaded with the tetracycline antibiotic embedded in carboxymethyl cellulose hydrogels. Additionally, the study used citric acid in order to avoid the cytotoxicity of conventional crosslinkers. The results showed that the addition of MCM-41 increased the capacity of antibiotic loading and prolonged its continuous release. Moreover, the system exhibited high antibacterial effects and cytocompatibility, proving its potential for wound healing and dressing systems [187]. Similarly, to further increase the antibacterial effects, one study reported the introduction of zinc oxide inside the MCM-41 nanoparticles [188].

However, there is a considerable public concern regarding non-regulated trace contaminants, which include pharmaceuticals, personal care products, illicit drugs, and halogenated flame retardants [189]. Among them, antibiotics represent an emerging environmental and health issue owing to their genotoxicity and mutagenicity and persistence in natural ecosystems, as they are continuously released into soils and surface and ground waters [189,190]. Antibiotics enter the environment via different routes, such as discharge of urban wastewater treatment or human waste, agricultural runoff, livestock farms waste through direct or indirect emissions owing to incomplete metabolism, discharge from drug manufacturers, and antibiotic abuse in aquaculture [189,191,192,193,194]. In terms of the effects on public health, antibiotic discharge could lead to endocrine disruption, toxicity of the unwanted and/or unknown byproducts, allergic reactions, and the development of antibiotic-resistant microorganisms and genes [189,195]. Antibiotics resistance has become one of the most important human health challenges of the 21st century. While there are many strategies applied for combatting the environmental sources of antibiotics, such as comprehensive monitorization and surveillance, hygiene programs, and wastewater treatment technologies, they are not able to efficiently remove antibiotics from the environment [196,197]. As even trace amounts can influence microbial populations and cause serious harm to the organism owing to cumulative effects [193,194], novel and low-cost technologies are needed to tackle such issues [197]. In this context, adsorption materials have been widely used for the process of water purification by conditioning, remediating, and removing inorganic and organic potentially harmful materials [190,197]. The molecular structure of antibiotics usually comprises a non-polar core and polar functional moieties that give their amphiphilic or amphoteric nature. However, their physicochemical properties vary considerably, which further induces differences in their adsorption behaviors. Therefore, the use of adsorptive materials for removing antibiotics from the environment has been reported for approximately 30 out of the ~250 antibiotics used [192,197]. Additionally, the sorption process must consider the environmental factors, including pH, metal ions, ionic strength, and organic matter [192].

In this regard, one study investigated the adsorption capacity of spherical MCM-41 towards quinolone antibiotics, namely enrofloxacin and norfloxacin. The results showed that the long alkyl chains of the templates enhanced the adsorption of the hydrophobic enrofloxacin, while having an inhibitory effect towards the hydrophilic norfloxacin [198]. Another study investigated the kinetics of the adsorption of norfloxacin on MCM-41 at a variety of pH, calcium ions concentration, ionic strength, and temperature. The results showed a maximum adsorption at the pH value of 7, and a high dependence on the electrostatic attractions and H-bond formations, as observed from the experiments at different ionic strengths and temperatures. Furthermore, it was shown that the adsorption at neutral pH is enhanced by non-electrostatic interactions such as hydrophobic and π–π associations. The presence of calcium ions increases the adsorption rate at pH values higher than 4.5 owing to the formation of ternary complexes through calcium-bridging. However, compared with other adsorptive materials, MCM-41 still has a low capacity of removing antibiotics from wastewaters, which suggests the need for further research and investigations [199]. Moreover, the adsorption capacity of ciprofloxacin hydrochloride using an iron-modified MCM-41 was investigated. The results showed that the highest removal efficiency was exhibited in the Si/Fe = 20 systems, at higher temperatures, with no impact of the pH value. Additionally, the systems proved stable performances for the use of four cycles [200]. Furthermore, the adsorption capacity of cephalexin using MCM-41 was tested through a Box–Behnken response surface methodology using the initial pH of the adsorbate, adsorbent dosage, initial concentration of the antibiotic, temperature, and contact time. The statistical analysis showed that the optimal conditions were pH 3, 800 mg/L of MCM-41, 50 mg/L of cephalexin, 40 °C, and 30 min of contact between the adsorbent and the adsorbate for a system with a pore diameter larger than 2 nm, a surface area of 1097 m^2^/g, and a crystallite size of 75 nm [201]. Considering the potential of MCM-41 and MSN to adsorb antibiotics within their pores, more studies should be performed for investigating the kinetics for such materials. In this manner, novel technologies that could help in reducing the persistence of antibiotics in the environment could be developed.

## 6. Conclusions

Over the past years, studies focused on MSN-based nanomaterials have extended their applications and clinical trials, with an emphasis on understanding their biocompatibility and cytotoxicity for biomedical applications. The most important characteristics of MSNs that have attracted scientific interest include tunable pore size, high surface area, large pore volume, and surface functionalization. Specifically, the presence of silanol groups on the surface of MSN allows for the variation of multiple properties, such as the hydrophobicity/hydrophilicity ratio, which makes them excellent candidates in a variety of applications, including industrial catalysis, absorbents, biosensors, drug delivery systems, gene carriers, phototherapy, and tissue engineering. Thus, the aim of this review was to highlight the main synthesis and functionalization strategies of the various MSN types and their most important applications in the medical, industrial, and environmental areas. Special attention was paid to reviewing the drug delivery systems, as well as to how these MSNs can be exploited in environmental applications, in removing antibiotics, pesticides, or other hazardous agents from the aqueous solutions. This aspect, even if it is not yet well developed in the literature, is of high importance because the presence of certain species in the wastewater, especially antibiotics, can lead to severe environmental issues and can induce antibiotic resistance for a wide range of microorganisms.

## Figures and Tables

**Table 1:** The characteristics of the main mesoporous silica nanoparticles (MSNs). MCM, Mobil Crystalline Materials or Mobil Composition of Matter; SBA, Santa Barbara amorphous; FSM, folded sheets of mesoporous materials; TUD, Technical Delft University; HMM, Hiroshima Mesoporous Material; COK, Centrum voor Oppervlaktechemie en Katalyse/Centre for Research Chemistry and Catalysis; FDU, Fudan University.

MSN Type	Dimensionality and Crystal System	Space Group	Pore Size [nm]	Surface Area [m^2^/g]	Pore Volume [cm^3^/g]	Reference
**MCM-41**	2D hexagonal	P6mm	1.5–8	900–2100	>1	[15,23,24,25]
**MCM-48**	cubic	Ia3d	1.5–6.5	900–1500	>1	[15,23,26]
**MCM-50**	lamellar	p2	2–5	n.a.	>1	[15,23,27]
**SBA-11**	cubic	Pm3m	2.1–3.6	n.a.	0.68	[15,23,27]
**SBA-12**	3D hexagonal	P6_3_/mmc	3.1	n.a.	0.83	[15,23,27]
**SBA-15**	2D hexagonal	p6mm	6–10	662	1.17	[15,23,28]
**SBA-16**	cubic	Im3m	5–15	1000	0.91	[15,23,27]
**FSM-16**	2D hexagonal	p6mm	3.2–3.9	500–900	0.96	[29,30,31]
**TUD-1**	disordered	-	2.5–25	300–1000	0.5–1.7	[32,33,34]
**HMM-33**	disordered	-	4–15	-	-	[35]
**COK-12**	hexagonal	P6m	5.5–6	860	0.45–1.23	[15,35,36]
**FDU-2**	cubic	Fd3m	2.3–3	960	0.98	[37]
**FDU-11**	tetragonal	P4/mmm	2.7	1490	1.88	[38]
**FDU-12**	cubic	Fm3m	36	250–450	0.27–0.48	[39]
**FDU-13**	orthorhombic	Pmmm	1.7	1210	1.83	[38]

n.a., information not available.

**Table 2:** The pH, silica precursor, surfactant, and additives required for the synthesis of MSN.

MSN Type	pH	Silica Precursor	Surfactant	Additives
**MCM-41**	basic	TEOS, TMOS	CTAB, cetyltrimethylammonium tosylate, cetyltrimethylammonium chloride, Pluronic F68	-
**MCM-48**	basic	TEOS	CTAB	-
**MCM-50**	basic	TEOS, TMOS	Gemini surfactants	
**SBA-15**	acid	TEOS, TMOS	Pluronic P123	-
**SBA-16**	acid	TEOS	Pluronic P123, Pluronic F127	potassium chloride
**FDU-12**	acid	TEOS	Pluronic F123	1,3,5-trimethylbenzene, xylene, toluene, potassium chloride
